# Parallel Structure from Motion for Sparse Point Cloud Generation in Large-Scale Scenes

**DOI:** 10.3390/s21113939

**Published:** 2021-06-07

**Authors:** Yongtang Bao, Pengfei Lin, Yao Li, Yue Qi, Zhihui Wang, Wenxiang Du, Qing Fan

**Affiliations:** 1 College of Computer Science and Engineering, Shandong University of Science and Technology, Qingdao 266590, China; zh_wang@sdust.edu.cn; 2 State Key Laboratory of Virtual Reality and Technology, Beihang University, Beijing 100191, China; linpengfei@buaa.edu.cn (P.L.); leeyao@buaa.edu.cn (Y.L.); qy@buaa.edu.cn (Y.Q.); 3 Virtual Reality Research Institute, Beihang University Qingdao Research Institute, Qingdao 266100, China; dwxiang@buaa.edu.cn; 4 MiningLamp Technology, Beijing 100102, China; fanqing@mininglamp.com

**Keywords:** structure from motion, graph segmentation, sparse point cloud, large-scale scene, camera clustering, UAV image

## Abstract

Scene reconstruction uses images or videos as input to reconstruct a 3D model of a real scene and has important applications in smart cities, surveying and mapping, military, and other fields. Structure from motion (SFM) is a key step in scene reconstruction, which recovers sparse point clouds from image sequences. However, large-scale scenes cannot be reconstructed using a single compute node. Image matching and geometric filtering take up a lot of time in the traditional SFM problem. In this paper, we propose a novel divide-and-conquer framework to solve the distributed SFM problem. First, we use the global navigation satellite system (GNSS) information from images to calculate the GNSS neighborhood. The number of images matched is greatly reduced by matching each image to only valid GNSS neighbors. This way, a robust matching relationship can be obtained. Second, the calculated matching relationship is used as the initial camera graph, which is divided into multiple subgraphs by the clustering algorithm. The local SFM is executed on several computing nodes to register the local cameras. Finally, all of the local camera poses are integrated and optimized to complete the global camera registration. Experiments show that our system can accurately and efficiently solve the structure from motion problem in large-scale scenes.

## 1. Introduction

Structure from motion (SFM) has rapidly developed in the field of 3D reconstruction. Image feature extraction and matching have generally achieved considerable success in computer vision. Until now, the existing research used a single computing node to reconstruct a three-dimensional (3D) sparse point cloud for small scenes with thousands of images as input. However, with the increase in the number of datasets, the reconstruction of large scenes must adopt distributed computing to ensure the accuracy and efficiency of reconstruction.

In the entire SFM step, image matching and epipolar constraint filtering are the most time consuming. To solve the problem of time consumption, Li et al. [1] used the principle of spatial angular order to improve efficiency, which assumes that angular order of neighboring points relating to one correspondence remains invariant under a variety of transformation. This constraint was used to remove outliers from initial matches of image pairs [2,3]. Some prior information of data acquisition can be used to achieve image pair selection without sacrificing computation costs. By leveraging the temporal consistency constraint, Aliakbarpour et al. [4] restricted feature matching to their forward and backward neighbors within a specified time offset. Considering that the long distance between the images is low or the images have no correlation, we use the global navigation satellite system (GNSS) information from the image to calculate the distance threshold of the GNSS effective neighbors and perform image matching for each image with its effective neighbors. The original squared time complexity of image matching is optimized for linear complexity.

Inspired by the divide-and-conquer framework, we propose camera clustering based on normalized-cut segmentation in this study. First, we divide the camera graph. The efficiency and robustness are weighed to perform the expansion work. We then delete the effective edges when graph cutting is restored. The clustering of cameras guarantees execution of the divide-and-conquer framework and preserves most of the connectivity between cameras. For merging blocks and reconstruction results of the subgraph, we select a reference camera and use it to calculate the scale ratio of the two blocks. The advantage of our system is that it only needs to meet two coincidence point pairs between the blocks—it does not require each block to have an overlapping relationship. The graph segmentation method can also solve the problem of global SFM sensitivity to outliers. The registration of global camera poses is completed through merger and optimization of the blocks.

In summary, this study makes the following contributions to existing literature:

- We propose a method for calculating the GNSS neighborhood, which greatly reduces the time of image matching and ensures the robustness of the matching relationship.
- We propose a distributed camera registration algorithm to ensure a strong correlation within the camera blocks and robustly merge and optimize all of the blocks to obtain an accurate global camera pose.
- Our algorithm is deployed in a distributed system that can ensure the efficiency and accuracy of large-scale 3D reconstruction work.

## 2. Related Work

### 2.1. Traditional SFM

The traditional SFM is divided into two methods: incremental SFM and global SFM. Incremental SFM [5,6,7,8,9,10,11] has been widely used in the first decade of SFM. It initializes image pairs and performs unified image extraction, matching, and epipolar constraint filtering. After obtaining the image relationship, it solves camera registration, triangulation, and bundle adjustment [12,13,14] to obtain a sparse point cloud. Furthermore, this method continues to add images and repeats the abovementioned operations to complete the final reconstruction. An incremental SFM method that continuously adds new images causes drift errors or generates only a part of the scene. Moreover, because each image is added to the calculation, bundle adjustment must be repeated multiple times to ensure the accuracy of the camera pose and the 3D point cloud. Therefore, the entire process consumes a considerable amount of time. The memory requirements are increasing, which proves disadvantageous for limited computing resources.

Compared with the incremental SFM, global SFM [15,16,17,18,19,20,21,22,23,24,25,26,27] shows better efficiency. It simultaneously calculates all camera poses from the available epipolar geometry and the trifocal tensor. After registering all camera poses, the global SFM method only executes bundle adjustment once at the end, which makes up for the lack of multiple incremental calculations. Global SFM uses image matching [28,29,30] and epipolar constraints [31,32,33] to generate an initial camera graph. First, it uses the relationship between the cameras to form a track. Next, it calculates the relative motions [34,35,36,37,38] between the cameras. Then, it performs global rotation and translation optimizations through these relative motions to obtain the camera global motions. Finally, global SFM performs triangulation and bundle adjustment to obtain the 3D sparse point cloud.

Global SFM is particularly dependent on the accuracy of image matching and is sensitive to outliers. Otherwise, its reconstruction integrity is not as good as that of incremental SFM. To counter this problem, hybrid SFM (HSFM) was introduced. HSFM uses the respective advantages of incremental SFM and global SFM to solve large-scale 3D reconstruction problems. It calculates the global camera rotation at once and incrementally retrieves the camera projection centers [39,40,41,42,43,44,45]. HSFM [27,44,45,46] proposes a divide-and-conquer framework that divides the camera graph. Then, it executes the reconstruction of the subgraph. However, a simple graph cut [47,48] reduces excessive image matching relationships. This results in an incomplete or incorrect integration of subgraph reconstruction.

### 2.2. Deep Learning-Based Reconstruction

The application of deep learning in geometric matching pipelines mostly focuses on local feature detection and descriptor learning [49,50]. Mismatch removal work based on deep learning [44,51] has also been well applied. In previous work [52], the Point-Net class architecture and context normalization were used to classify the inferred correspondence. However, this study could not take advantage of the relative motion pose shared by neighboring pixels [53]. Zhang et al. [54] used neural networks to infer the probability of each corresponding point as an interior point and then restored the camera pose. Tabb et al. [55] used rigid constraints to represent the camera network and multi-camera calibration problem and expressed it as a system of equations to obtain approximate solutions. Liu et al. [56] developed a new type of deep neural network (LPD-Net), which can extract distinguishable global descriptors from the original 3D point cloud. Yao et al. [57] proposed a multi-view-based depth map understanding framework (MVSNet) for the 3D reconstruction of outdoor scenes. Their method only calculates one depth map at a time instead of calculating the entire 3D scene. Gu et al. [58] further improved MVSNet, which solved the cubic increase in computational complexity as the image resolution increased. Most 3D reconstruction algorithms are only applicable to static scenes. Miksik et al. [59] proposed an end-to-end system for the real-time reconstruction of outdoor dynamic environments. The system operates in an incremental manner and can process scenes of objects in real time. For the application of outdoor dynamic scenes, Hu et al. [60] further proposed an efficient and lightweight network to directly determine the semantics of each point in a large-scale point cloud. In short, the current deep learning-based methods can process low-resolution input image sets. They are mostly suitable for indoor scenes or small-scale outdoor scene reconstruction work. Therefore, the problem of sparse point cloud reconstruction for large-scale scenes requires the use of geometric information-based motion recovery structure technology.

### 2.3. Large-Scale SFM

Some studies attempted to solve large-scale SFM through multi-core computing nodes [5,21,61,62] or by reducing the time of pair matching [28,29] through construction of skeleton diagrams [62,63,64]. Bhowmick et al. [46] attempted to solve the large-scale SFM problem in a divide-and-conquer manner. They used the graph cut [47,48] to divide the camera graph. After all of the sub-graph reconstructions are completed, other cameras are registered in each subgraph to construct overlapping areas and to merge them. This method was improved in [27,65] to cluster the dataset and to merge each cluster through a distributed camera model. However, both [27] and [46] did not consider the graph clustering strategy well. The study conducted by [66] ignored the careful design of clustering and merging algorithms, which made the reconstruction fragile and caused drift errors.

With the rapid increase in the number of relative motion calculations [34,35,36,37,38], the standard motion average problem that considers all relative postures simultaneously becomes both memory-intensive and time-consuming. This problem becomes more obvious in the translation averaging [19,20,31,62,67] that considers the relative translation between the camera and the 3D point.

## 3. Methodology

### 3.1. Overview

To solve the problem of large-scale motion averaging in a distributed way, we propose a divide-and-conquer framework to complete global camera pose registration in a distributed manner. We also introduce GNSS information as a filter criterion for image matching, as it can reduce the time complexity of the image matching part of the traditional SFM method to linear time complexity.

**Figure 1:**
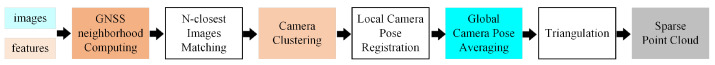
Pipeline of the proposed system.

Figure 1 shows the pipeline of our method. Given the collection of images and their features from large-scale scenes with unmanned aerial vehicle (UAV), our method can effectively generate sparse point cloud using GNSS neighborhood, camera clustering, and global camera pose averaging. The images and their features are considered as inputs. The GNSS neighborhood is calculated for image matching. Camera clustering and local camera pose registration are used for global camera pose averaging. Executing triangulation and optional bundle adjustment can obtain sparse point clouds. Specifically, our method consists of the following three steps: GNSS neighborhood computing, camera clustering, and camera pose averaging.

### 3.2. GNSS Image Matching

Image matching [28,29] is a key step in SFM. The accuracy of the matching relationship affects the accuracy of the final camera pose and the integrity of the reconstruction results. Reconstructing large scenes requires a huge number of high-resolution images as input. The traditional image matching has a square-level time complexity, which takes up more than half of the time in the entire SFM pipeline. We use GNSS information to calculate the GNSS neighborhood as the filter conditions for image matching. The time complexity of image matching is optimized to be approximately linear.

#### 3.2.1. GNSS Neighborhood Computing

WGS84 to ECEF coordinate system conversion: The GNSS system uses position satellites to locate and navigate in real time. Almost all cameras used in UAVs have the possibility of storing the drone’s GNSS information inside the exchangeable image file (EXIF) of the image. GNSS coordinates are based on the World Geodetic System 1984 (WGS84). We mark the GNSS coordinates in the WGS84 coordinate system as $P_{G}={\left[\lambda ,\varphi ,h\right]}^{T}$, where λ represents longitude, φ represents latitude, and *h* represents height, which is the height from the surface of the ellipsoid. As GNSS coordinates uses the WGS84 ellipsoid as a reference surface, we need a Cartesian 3D model to correctly calculate the distance between the two cameras in space. We need to convert the WGS84 coordinate system to the Earth-centered Earth-fixed (ECEF) coordinate system [68]. The conversion method is as follows:

semi−majoraxis:a=6378137,

semi−majoraxis:b=a(1−f),

flattening:f=1/298.257223563,

$$eccentricity:e=\sqrt{\frac{a^{2}-b^{2}}{a^{2}}},$$

where *a* and *b* represent the length of Earth ellipsoid’s semi-major axis and semi-minor axis in the geodetic system, respectively; *f* represents the flattening factor of the earth; and *e* represents the eccentricity of the Earth.

(1) $$P_{E}={\left[\left(N+h\right)cos\varphi cos\lambda ,\left(N+h\right)cos\varphi sin\lambda ,\left[N\left(1-e^{2}\right)+h\right]sin\varphi \right]}^{T},$$

where the variable $P_{E}$ is the image coordinate in the ECEF coordinate system and *N* is defined as $N=\frac{a}{\sqrt{1-e^{2}sin^{2}\varphi }}$.

Spatial threshold calculation: Given the input *N* images, we calculated the spatial Euclidean distance between $\frac{N(N-1)}{2}$ pairs of images. We recorded the coordinates of the two images as $P_{1}\left(x_{1},y_{1},z_{1}\right)$ and $P_{2}\left(x_{2},y_{2},z_{2}\right)$, respectively. The formula for calculating the spatial Euclidean distance is as follows:

$$distance_{\left(P_{1},P_{2}\right)}=\sqrt{{\left(x_{1}-x_{2}\right)}^{2}+{\left(y_{1}-y_{2}\right)}^{2}+{\left(z_{1}-z_{2}\right)}^{2}}.$$

The farthest distance maxD of each image can be obtained by sorting the GNSS coordinate distance of each pair of images. To ensure the efficiency and robustness of the system reconstruction, the distance *D* is calculated such that each image is matched with other images in less than the distance $D_{threshold}$.

(2) $$D_{threshold}=\frac{maxD}{\sqrt{N}}\delta_{l},$$

where $\delta_{l}$ is the scale constant. We set it as 10 in our experiments.

#### 3.2.2. Image Matching

Pre-matched view graph establishment: After obtaining each image and all GNSS neighbors that meet the distance threshold, we generated a pre-matched camera graph structure based on the GNSS neighborhood. First, we selected the set of hash functions according to the locality sensitive hash (LSH) algorithm [69] and randomly selected *m* hash functions. We then used scale-invariant feature transform (SIFT) for feature points detection and mapped the image feature points to a hash table, which contains $2^{m}$ different bits hash bucket. If there is a matching relationship between the two images, the number of feature points in each hash bucket obtained by using the same hash function set *H* should be similar. Finally, we selected multiple hash function sets $\left\{H|H_{1},H_{2},\ldots H_{n}\right\}$ and mapped the input images to multiple hash tables. The images in each hash bucket form a matching relationship. We restricted the number of matching times *k* for each image to improve the time efficiency of image matching. In our experiment, we added the matching relationship to the pre-matched map only when the *k* of two images is both less than 100. The image matching and epipolar geometric constraint filtering are performed according to the pre-matching map.

Cascade hashing image matching: We used the LSH algorithm to implement a cascade hashing image matching method. This method includes three steps: rough query, mapping Hamming space, and hash sorting. First, we recorded the feature points in all hash tables that fall into the same hash bucket and used these hash buckets as the scope of the query. We then mapped each feature point to a higher dimensional Hamming space (128 dimension in our experiment) and only calculated the descriptor similarity between the feature points for which the Hamming distance is less than the threshold. Finally, the problem was transformed into a topk problem of finding the smallest Hamming distance among candidate feature points. We adopted the idea of hash sorting and used the Hamming distance as the key to establish a hash bucket. Points with the same Hamming distance as the query point fall into the same bucket.

Epipolar constraint: We first obtained all matching relations that were accurate and satisfied the epipolar geometry. We then established the camera graph G=(V,E), where *V* represents the cameras and *E* is the matching relationship between the cameras. The vertex $v_{i}\in V$ in the GNSS neighborhood represents a camera that is an image. The edge $e_{ij}\in E$ among vertices indicates that the distance between the vertices meets the threshold $D_{threshold}$. The weight of the edge $w_{ij}$ represents the number of matching feature points $M_{ij}$ between the two images, that is, $w_{ij}=\left|M_{ij}\right|$.

### 3.3. Camera Clustering

With the increasing number of input images for the SFM, two problems need to be solved. The first is that the program exceeds the memory limit of a single computing node. Second, the parallelized computer cluster resources are difficult to use [5,21,61,62]. Therefore, we divided the camera graph into multiple subgraphs and utilized multiple computing nodes in parallel to solve the above two problems.

The segmentation of the camera graph should be divided into two steps, namely division and expansion. Division enables each sub-problem to solve the *enforceability constraint* on a single computing node. The expansion makes all adjacent subgraphs overlap enough to meet the *consistency constraint*. This ensures that the corresponding poses are merged in motion averaging. At the same time, accurate global camera poses and complete reconstruction results can be obtained.

#### 3.3.1. Normalized-Cut Algorithm

We use a normalized-cut algorithm to divide the camera graph. The matching relationship among the images was used as the weight. The camera graph was divided into multiple subgraphs. As there needs to be a coincidence point between subgraphs, it is necessary to select the edge with the greatest weight from the edges removed by the division. We retrieved this from each subgraph.

The normalized-cut algorithm is a graph segmentation algorithm. A weighted undirected graph G=(V,E) can be divided into two non-connected subgraphs, *A* and *B*. This can be done by deleting some edges so that A∪B=V,A∩B=∅. The sum of the weights of the removed edges is regarded as the dissimilarity of the two parts, *A* and *B*. It can further be defined as $cut\left(A,B\right)=\sum_{u\in A,v\in B}w(u,v)$. The sum of the weights of all nodes in subgraph *A* and all of the nodes *V* in graph *G* can be recorded as $assoc\left(A,V\right)=\sum_{u\in A,v\in V}w(u,v)$, where w(u,v) is the weight of the edge. The normalized segmentation method [48] is given as follows:

(3) $$Ncut\left(A,B\right)=\frac{cut(A,B)}{assoc(A,V)}+\frac{cut(B,A)}{assoc(B,V)}.$$

Note that minimizing the calculation result ensures that the subgraphs have a small degree of correlation and that the subgraphs have a large degree of correlation.

#### 3.3.2. Camera Graph Division

We used G=(V,E) to represent the camera graph, where each vertex $v_{i}\in V$ represents a camera $c_{i}\in C$ and each edge $e_{ij}\in E$ has a weight $w_{e_{ij}}$ to connect two different cameras, $c_{i}$ and $c_{j}$. We used the number of matching feature points between the two images to represent the weight, expressed as $w_{e_{ij}}=\left|M_{ij}\right|$. The camera graph *G* is divided into several subgraphs $G_{i}$ such that each subgraph is controlled within its size. The number of vertices in each subgraph was similar. All of the divided subgraphs were recorded as the set $G_{c}$. The enforcement constraint needs to satisfy

(4) $$\begin{array}{c} \forall G_{i}\in G_{c},\left|V_{i}\right|\leq N_{limit},\\ \forall G_{i},G_{j}\in G_{c},\left|V_{i}\right|\approx \left|V_{j}\right|, \end{array}$$

where $N_{limit}$ represents the maximum number of cameras in each subgraph, $G_{i}$ and $G_{j}$ represent the subgraph, and $\left|V_{i}\right|$ and $\left|V_{j}\right|$ represent the number of vertices in the subgraph of $G_{i}$ and $G_{j}$. The normalized segmentation ensures that the segmented subgraph meets $\left|V_{i}\right|\approx \left|V_{j}\right|$. $N_{limit}$ affects the calculation time of the local SFM, multi-block expansion, and data transmission time of computing nodes. We chose $N_{limit}=2000$ to deal with large-scale scene reconstruction based on the reconstruction effect and efficiency. Our predecessors [44] used 100 for $N_{limit}$. However, this causes many graph segmentation operations and data transmission, and the local bundle adjustment cannot cover the number of cameras. More subgraph merging also causes a large drift error. We set $N_{limit}$ as 2000 so that each computing node can process and obtain more accurate reconstruction results.

#### 3.3.3. Camera Graph Expansion

Each subgraph must have overlapping vertices to complete the camera pose synthesis and subsequent reconstruction work between subgraphs. Therefore, the subgraphs after division must be expanded accordingly. Each subgraph does not need to overlap with all of the other subgraphs. However, the coincidence ratio between the subgraphs must be guaranteed to ensure that each subgraph can be merged. This is not affected by the relationship between the subgraphs. We define the consistency constraint as

(5) $$\forall G_{i}\in G,\frac{\left|V_{expansion}\right|}{\left|V_{i}\right|}\geq \delta_{ratio},$$

where $V_{expansion}$ represents the vertices of expansion and $\delta_{ratio}$ is the expansion ratio. The edges deleted during the division of each subgraph were sorted by weight, according to the number of matching feature points. After that, we obtained some edges with the largest weight. The two vertices of the edge were added to the subgraph, and the number of these points is $\left|V_{expansion}\right|$. We set the $\delta_{ratio}$ to 0.5 in our experiments. We stopped the expansion when the ratio of the expanded vertices was greater than or equal to this ratio, or all of the cut edges between the subgraphs were restored.

When all of the removed edges of multiple subgraphs were restored, the overall expansion ratio was lower, resulting in insufficient matching relationships. In this case, we performed a secondary expansion on all subgraphs. We defined the ratio of all new vertices to the total number of original vertices as the expansion consistency constraint as

(6) $$\frac{\sum \left|V_{\exp ansion}\right|}{\sum \left|V_{i}\right|}\geq \delta_{ratio},$$

where $\delta_{ratio}$ denotes the same meanings as in Equation (5). If the condition is not satisfied, the edges discarded by the normalized-cut segmentation are selected according to the weights in descending order. We then randomly add the new edge to one of the two blocks connected by the new edge, and then iteratively expand until the expansion ratio is satisfied. This ensured that there were enough coincidence points between each subgraph. Moreover, this could restore a large number of connections through the expansion of all subgraphs to ensure the accuracy of the integration result and avoid wasting computing resources due to the redundancy of the extended cameras.

### 3.4. Camera Pose Averaging

The camera poses in each subgraph can be obtained by executing the local SFM for each subgraph. In this section, we provide a fast and accurate averaging method to merge and optimize the previously calculated camera poses of each subgraph. We obtain global camera poses for subsequent triangulation and bundle adjustment.

A similar transformation method to that in [34] was used to merge the camera pose. The conversion formula for any two camera poses is as follows:

(7) $$\left[\begin{array}{cc} R_{j} & T_{j}\\ 0^{T} & 1 \end{array}\right]=\left[\begin{array}{cc} r_{ij} & t_{ij}\\ 0^{T} & 1 \end{array}\right]\left[\begin{array}{cc} R_{i} & T_{i}\\ 0^{T} & 1 \end{array}\right],$$

where $R_{i}$ and $T_{i}$ are the rotation and translation of camera *i*, respectively; $R_{j}$ and $T_{j}$ are the rotation and translation of camera *j*; and $r_{ij}$ and $t_{ij}$ are the relative rotation and translation between camera *i* and *j*, respectively. $R_{j}$ and $T_{j}$ can be derived as

(8) $$\begin{array}{c} R_{j}=r_{ij}R_{i},\\ T_{j}=r_{ij}T_{i}+t_{ij}. \end{array}$$

These equations represent the conversion relationship between the poses of the two cameras. The camera poses in the world coordinate system of the two cameras can be used to estimate the relative rotation and translation between the two cameras. The camera pose was merged using a similar transformation method. We optimized the merged camera pose by nonlinear optimization to obtain an accurate global camera pose.

#### 3.4.1. Global Rotation Registration

We denote the repeated cameras $C_{rpt}$ in any two clusters $C_{i}$, $C_{j}$ as $\left\{C_{rpt}|C_{rpt}=C_{i}\cap C_{j}\right\}$. We first calculated the relative rotations $r_{rel}$ of the coincident point and all points in the cluster that need to be transformed. We then fixed the global rotations of the repeated cameras. The original camera rotations $R_{rpt}$ in $C_{j}$ were updated to the corresponding $R_{rpt}^{{}^{\prime }}$ of the same camera in $C_{i}$. Finally, the updated global rotation $R_{j}^{{}^{\prime }}$ of the other cameras in the cluster was obtained as follows:

(9) $$\forall c_{j}\in C_{j},R_{j}^{{}^{\prime }}=r_{ij}R_{rpt}^{{}^{\prime }}.$$

Except for the fixed camera pose, all other camera poses belonging to the subgraphs need to be calculated. This results in some coincidence points that are repeatedly calculated for the pose. These repeated calculations were used as a benchmark for error. We first selected a fixed camera pose, which we believed was the most accurate reference camera. We then set the error between the camera poses of all calculated coincidence points and the original pose as the smallest one. Finally, we used this camera as the base of the camera’s global rotation registration.

#### 3.4.2. Global Translation Registration

The rotations and translations of the camera poses in each subgraph have their own scales. These scales do not affect the abovementioned method of global rotation registration. However, the registration of the global translation registration needs to calculate the difference scale value $\lambda_{t}$ between the local translation in each subgraph scale.

As shown in Equation (7), without considering the scale of the same subgraph, the conversion relationship between the local translations of the two cameras is as follows:

(10) $$\begin{array}{c} T_{2}=r_{12}T_{1}+t_{12},\\ T_{2}^{{}^{\prime }}=r_{12}T_{1}^{{}^{\prime }}+t_{12}^{{}^{\prime }}, \end{array}$$

where $T_{1}^{{}^{\prime }}$ and $T_{2}^{{}^{\prime }}$ are the translations of cameras 1 and 2 in subgraph 1, $T_{1}^{{}^{\prime }}$ and $T_{2}^{{}^{\prime }}$ are the translations of cameras 1 and 2 in subgraph 2, and $t_{12}$ and $t_{12}^{{}^{\prime }}$ are the relative translations between cameras 1 and 2 in subgraphs 1 and 2, respectively. We defined the pairs of repeated cameras in the two subgraphs to be merged as $\left\{T_{1},T_{2}\right\}$ and $\left\{T_{1}^{{}^{\prime }},T_{2}^{{}^{\prime }}\right\}$. As the translation of the same camera in two different coordinate systems is to be calculated, $r_{12}$ remains the same.

The camera poses in each subgraph can be obtained by executing the local SFM for each subgraph. The relationship between the local translation within two subgraphs can be obtained after executing the local SFM. We combined all of the coincident points $C_{rpt}$ into $\frac{n(n-1)}{2}$ pairs, where $n=\left|C_{rpt}\right|$. We then obtained the most accurate scale $\lambda_{t}$ according to the following formula:

(11) $$\lambda_{t}=\frac{\sum_{i,j\in \{C_{rpt}\}}\frac{\left|t_{i{}^{\prime }j{}^{\prime }}\right|}{\left|t_{ij}\right|}}{\left|C_{rpt}\right|},$$

where $\frac{\left|t_{i{}^{\prime }j{}^{\prime }}\right|}{\left|t_{ij}\right|}$ is the scale ratio calculated according to each group of camera pairs.

In the global rotation registration, we selected the most accurate reference camera, and in the global translation registration work, we used the same camera as the reference. We marked the translation of the reference camera in the current coordinate system and the target coordinate system as $T_{a}$ and $T_{a}^{{}^{\prime }}$, and the translations of the camera to be merged in the current and target coordinate system were denoted by $T_{b}$ and $T_{b}^{{}^{\prime }}$ respectively. $r_{ab}$ represents the relative rotation of the reference camera and other cameras. The relevant formula for the camera translation is given as

(12) $$t_{ab}=T_{b}-r_{ab}T_{a},$$

(13) $$T_{b}^{{}^{\prime }}=r_{ab}T_{a}^{{}^{\prime }}+\lambda_{t}t_{ab}.$$

We first calculated the relative translation $t_{ab}$ between the camera translation $T_{b}$ and the reference camera translation $T_{a}$ using Equation (12). We then converted the reference camera translation $T_{a}$ into translation $T_{a}^{{}^{\prime }}$ in the target coordinate system. Finally, we used the calculated translation scale ratio $\lambda_{t}$ to calculate the global translation $T_{b}^{{}^{\prime }}$ in the target coordinate system according to Equation (13).

In this section, we discuss how the camera pose is merged between the two sub-images. We can also merge new subgraphs through continuous iterations and finally obtain a unified global camera pose. Our method does not require overlap between each subgraph; it only needs to ensure that there is sufficient overlap relationship, so that a new subgraph can be merged repeatedly through iteration. As each new subgraph only needs to have an overlap relationship with the merged group of pictures, the number of overlapping points is at least 2.

#### 3.4.3. Optimization of Camera Poses

Calculation of the translation scale among the coordinate systems can be guaranteed to be accurate by averaging it with the selections from the reference camera. A relatively complete reconstruction result can be obtained by directly applying these camera poses to the subsequent reconstruction work. However, the details of the reconstruction result are greatly reduced. In this section, we give more consideration to the relationship between the sub-images and further optimize the previously calculated camera pose to obtain a more refined reconstruction result. We keep the relative rotations and translations obtained when local SFM is executed on each subgraph and record them as $R_{rel}$ and $T_{rel}$, respectively. After the global rotation and translation registration, these relative relationships are merged, which can cross-influence the entire synthesized camera pose. Therefore, we use the following method to optimize the previously registered global camera pose $\gamma =\left\{R_{i}\right\},\tau =\left\{T_{i}\right\}$.

(14) $$\begin{array}{c} \arg\min_{\gamma }\sum_{r_{ij}\in R_{rel}}d^{R}{\left(r_{ij},R_{j}R_{i}^{T}\right)}^{P},\\ \arg\min_{\tau }\sum_{t_{ij}\in T_{rel}}d^{T}{\left(t_{ij},T_{j}-R_{j}R_{i}^{T}T_{i}\right)}^{P}, \end{array}$$

where $r_{ij}$ and $t_{ij}$ denote the same meanings as in Equation (8), $d^{R}$ represents the chordal distance, $d^{T}$ represents the Euclidean distance, and *P* takes 2 to represent the $L_{2}$ normal form.

These local camera poses are obtained from each subgraph, which can ensure that the relationship between them is accurate because of the advantage of normalized-cut. Therefore, we used the camera pose as input to avoid calculating the relationship among all camera poses. This can also completely optimize the overall global camera pose. After optimizing the above camera pose, a robust and accurate global camera pose was obtained for subsequent triangulation, and hence, a sparse point cloud with an accurate position could be obtained.

## 4. Results

### 4.1. Implementation Details

We implemented GNSS neighbor computing, camera clustering, and global camera pose merging and optimization on a single computer. The server system was Ubuntu 16.04, g++ 9.2.0 and was configured with an Intel(R) Xeon(R) CPU E5-2680 v4 at 2.40 GHz 28-core 96 GB memory. We also performed image matching and local camera pose estimation on a distributed computing system consisting of eight computers. Table 1 summarizes the cluster configuration experimental environment, including eight computers, of which the 28-core 96 GB memory computer is the master node and the remaining computers are the slave nodes. All of the computers were deployed on a scalable network file system similar to the Hadoop File System. The large-scale datasets used in this study are all drone aerial images with a resolution of 2736 × 1824.

### 4.2. GNSS Neighbor Computing and Image Matching

**Figure 2:**
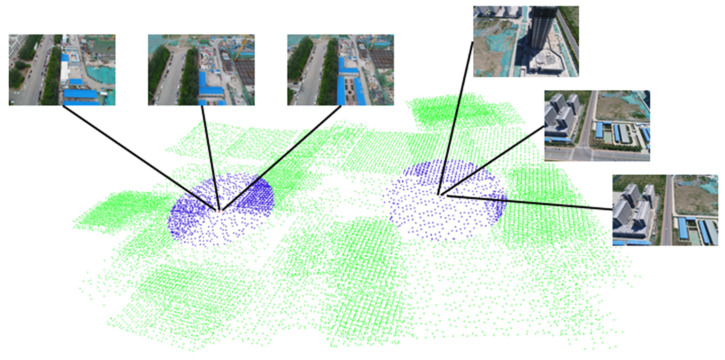
GNSS neighbor computing result of Tianjin Xuefu Industrial Zone.

In this section, we use three aerial datasets to validate our method. Aerial dataset 1 is an area of Tiangong University, which contains 1510 images. Dataset 2 is the entire campus of Tianjin University of Technology and contains 8876 images. Dataset 3 is the Tianjin Xuefu Industrial Zone, which contains 25,726 images. Figure 2 shows the GNSS neighbor computing results of dataset 3. In this figure, two cameras are sampled and marked in red, and their respective GNSS neighbors are marked in blue. The camera represented by the red dot only performed image matching with the blue dot camera, avoiding redundant image matching with the green dot cameras. This can greatly reduce the time required for image matching.

To verify the effectiveness of our image matching method, we performed a time-consuming analysis on these three datasets. Table 2 presents the time required for image matching using the traditional method, our method on a single computer, and our method on a cluster. It takes time to transmit images and feature points among clusters, and there are fewer matching pairs filtered by GNSS information. The optimization effect is not obvious on dataset 1 owing to a small number of images. As our method reduces the time complexity of image matching from square to approximately linear, our method on a cluster can show good optimization effects on datasets 2 and 3. Performing an image matching experiment on dataset 2, the result of our method on the cluster was approximately 16 times faster than the conventional method. The image matching speed of our method was approximately 55 times faster than that of the traditional method on dataset 3.

**Figure 3:**
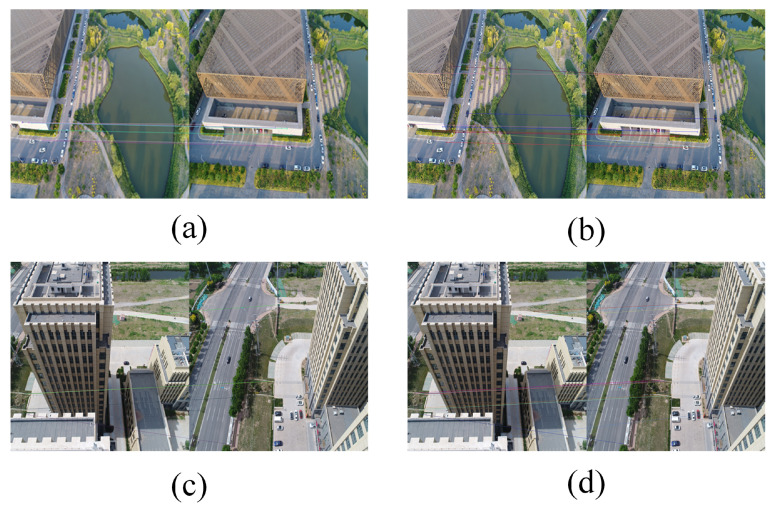
Comparison of image matching results. (**a**,**c**) The results using a traditional method such as SIFT. (**b**,**d**) The corresponding results generated by our cascade hashing image matching method.

Figure 3 shows a comparison of the image matching results generated by the traditional method and our cascade hashing image matching method. It can be seen from this figure that our method can obtain more matching points in a short time and that the quality of the matching points is higher, which can better represent the local features of images.

### 4.3. Camera Clustering Results

**Figure 4:**
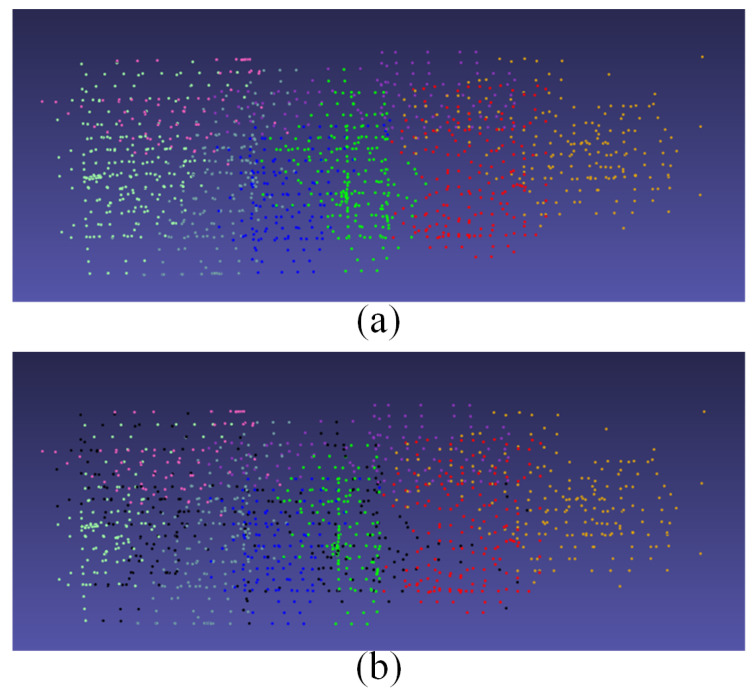
Camera graph division and expansion of an area for Tiangong University. Cameras in different colors in (**a**) represent different categories, and black cameras in (**b**) represent cameras that overlap between sub-images after camera expansion.

We used a single computer configuration to verify the accuracy of the camera clustering results. Figure 4 shows the camera graph division and expansion of the area of Tiangong University. The dataset contains 1510 UAV aerial images with a resolution of 2736 × 1824, where each point represents a camera. As shown in Figure 4, our method can enable effective camera division and expansion on medium-scale scenes.

**Figure 5:**
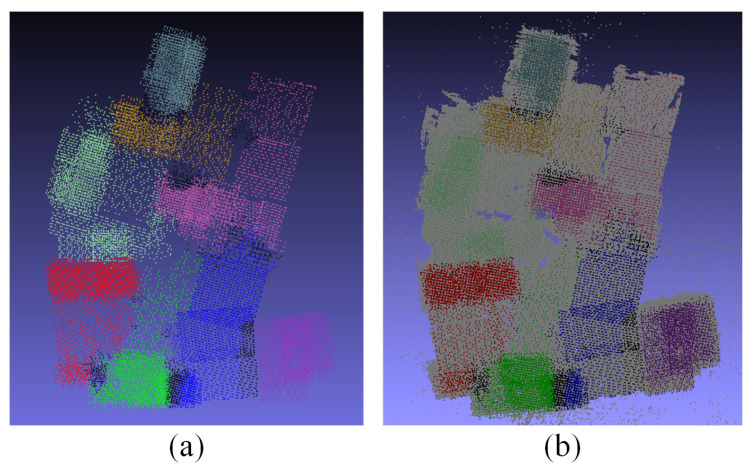
Camera clustering and sparse point cloud results of Tianjin Xuefu Industrial Zone: (**a**) different camera categories; (**b**) camera clustering and corresponding sparse point cloud.

**Figure 6:**
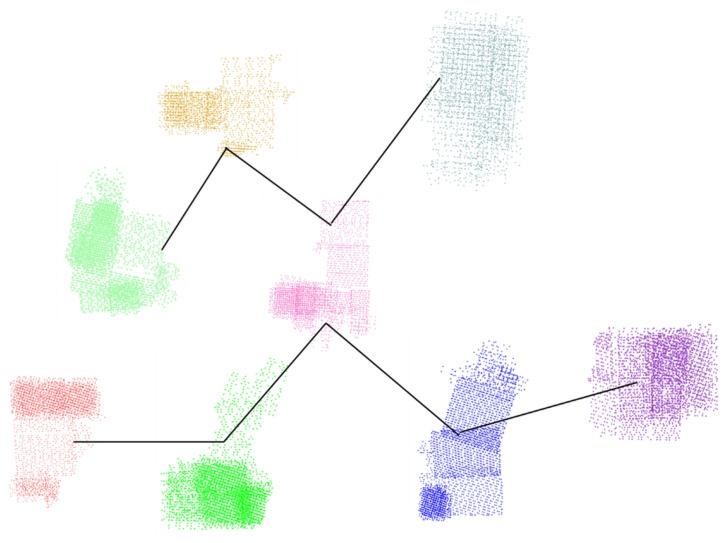
Maximum spanning tree composed of camera subgraphs.

We further applied our method to a large-scale scene to verify the effectiveness of the method. Figure 5 shows the camera clustering and sparse point cloud reconstruction results of the Tianjin Xuefu Industrial Zone. The dataset contains 25,726 UAV aerial images. Cameras with different colors indicate different camera clustering results. As shown in Figure 5, our method can be applied to camera clustering in large-scale scenes and subsequent sparse point cloud generation work. Figure 6 shows the maximum spanning tree composed of camera subgraphs in the Tianjin Xuefu Industrial Zone. The cameras of different colors correspond to the camera classification results in Figure 5. The edge weights connected between nodes are defined as $\frac{cut(A,B)}{\left|V_{A}\right|+\left|V_{B}\right|}$, cut(A,B), $\left|V_{A}\right|$, and $\left|V_{B}\right|$ and have the same meaning as in Section 3.3. We performed camera image expansion work according to the maximum spanning tree.

### 4.4. Camera Pose Estimation

As described in Section 4.1, global camera pose merger and optimization are performed by a single computer, and the local camera pose estimation step uses cluster resources to calculate the local camera pose of each camera sub-image in parallel.

**Figure 7:**
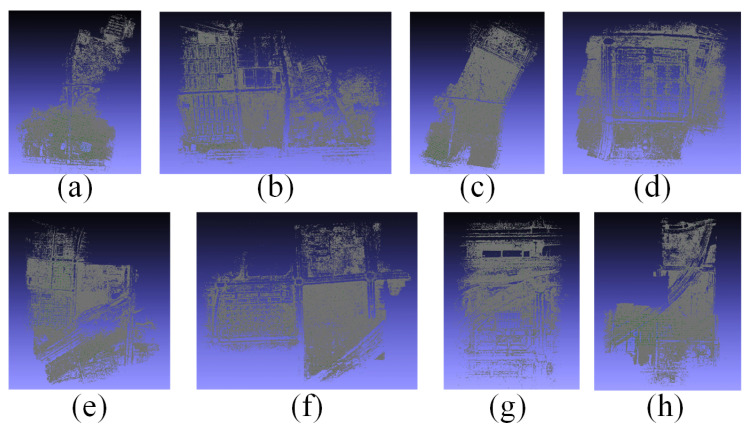
Local camera pose estimation results of the Tianjin Xuefu Industrial Zone. (**a**)–(**h**) show the different parts of the Tianjin Xuefu Industrial Zone.

**Figure 8:**
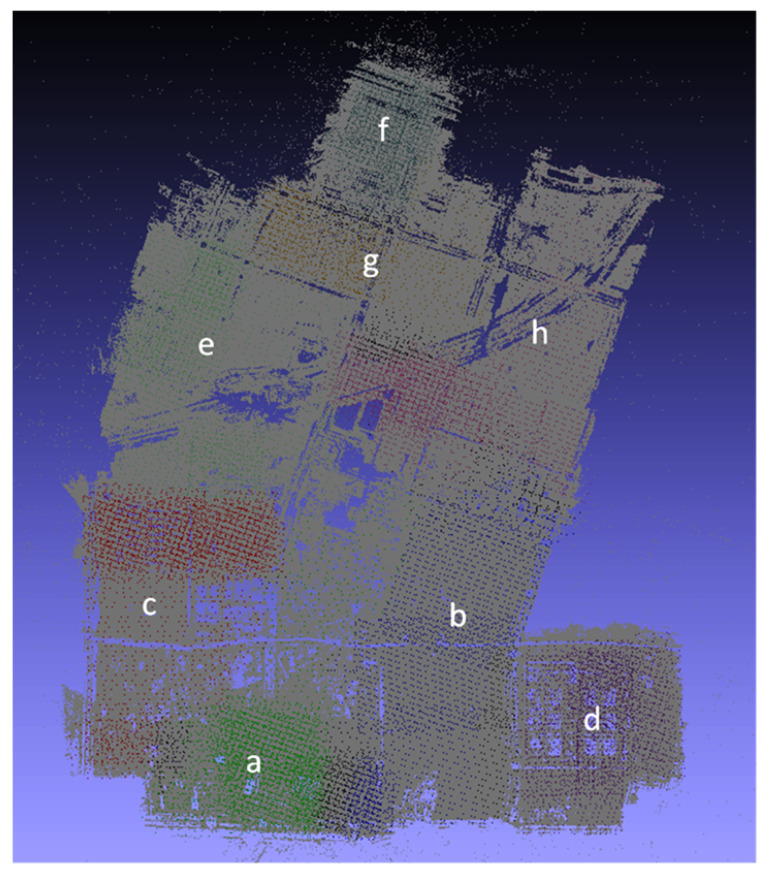
Local camera pose merging results of the Tianjin Xuefu Industrial Zone.

Figure 7 shows the results of the local camera pose estimation performed in parallel by each computing node of the Tianjin Xuefu Industrial Zone dataset. This result is displayed in the form of a sparse point cloud. We used the camera pose of each sub-image in the actual work. Figure 8 shows the final result of performing global camera pose registration after local camera pose estimation of each subgraph in Figure 7. The white-marked letters on the image correspond to the specific positions of the camera poses of each sub-image in the global space.

## 5. Discussion

We compared our approach with state-of-the-art sparse point cloud generation methods in different scenarios. We carried out experimental verification on the two types of internet public datasets and large-scale datasets.

### 5.1. Internet Public Datasets

**Figure 9:**
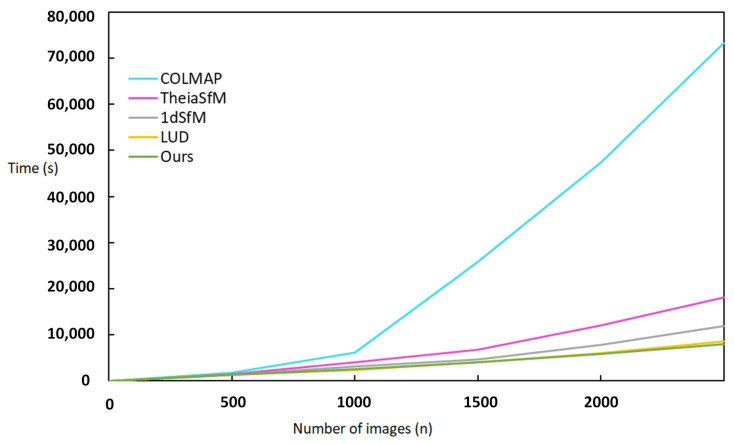
Efficiency evaluation on datasets with different algorithms.

Time efficiency evaluation: We compared our approach with two incremental SFM (COLMAP [9] and TheiaSfM [70]) and two global SFM (1dSfm [62] and LUD [26]) methods to measure the efficiency of our algorithm. As the public internet datasets are small, the comparison experiments were run on a single computer for the sake of fairness. Figure 9 shows the time-efficiency comparison of datasets with different algorithms. As observed from Figure 9, owing to the continuous implementation of bundle adjustment, the incremental SFM methods become very time-consuming as the number of images increases. Our approach completely adopts the global SFM methods, which reduce the time of bundle adjustment through the reconstruction of camera clustering and reduce the time of image matching through the GNSS neighbor matching mode. Our approach is proven to be faster than all current public methods when using a distributed system with a cluster configuration.

Accuracy and performance evaluation: To verify the accuracy and efficiency of our approach, we compared our method with several traditional methods on public internet datasets. Bundle adjustment is generally used as a final step in generating sparse point clouds. It is known as the estimation involving minimizing the reprojection error. Consider a situation in which a set of 3D points $P_{j}$ is viewed by a set of cameras with matrices $R^{i}T^{i}$; we denote by $x_{j}^{i}$ the coordinate of the *j*th point as seen by the *i*th camera. If the image measurements are noisy then the equations $x_{j}^{i}=R^{i}T^{i}P_{j}$ are not satisfied exactly. We estimate projection matrices ${\hat{R}}^{i}{\hat{T}}^{i}$ and 3D points ${\hat{P}}_{j}$, which project exactly to image points ${\hat{x}}_{j}^{i}$ as ${\hat{x}}_{j}^{i}={\hat{R}}^{i}{\hat{T}}^{i}{\hat{P}}_{j}$, and minimize the image distance between the reprojected points and detected image feature points $x_{j}^{i}$ for every view in which the 3D points appears. The reprojection error can be expressed as follows:

$$\min_{{\hat{R}}^{i}{\hat{T}}^{i},{\hat{P}}_{j}}\sum_{i,j}d{\left({\hat{R}}^{i}{\hat{T}}^{i}{\hat{P}}_{j}-x_{j}^{i}\right)}^{2},$$

where d(x,y) is the geometric image distance. The smaller the projection error of the control points, the higher the quality of the generated point clouds. In addition to the reprojection error, we also use the number of recovered cameras and the number of generated 3D point clouds to measure the accuracy of various methods. Table 3 presents the comparative experimental results of local camera pose estimation with COLMAP [9], 1dSfm [62], and LUD [26] on public internet datasets. The six public datasets in Table 3 are Courtyard, Aos Hus, Buddha, Cathedral, Palace, and Forum [71,72]. $N_{c}$, $N_{p}$, and $T_{\sum }$ represent the number of recovered cameras, the number of 3D points, and the reconstruction time, respectively. As the datasets are small, we gave up the advantages of a distributed system and only compared our approach with other methods in the local camera pose estimation. As shown in Table 3, our method can always recover most camera poses and sparse point cloud results at a faster speed.

In the final generation of sparse point cloud, we evaluated our algorithm on several public datasets [71,72]. For these small-scale internet public datasets, we ran our system on only one computer.

**Figure 10:**
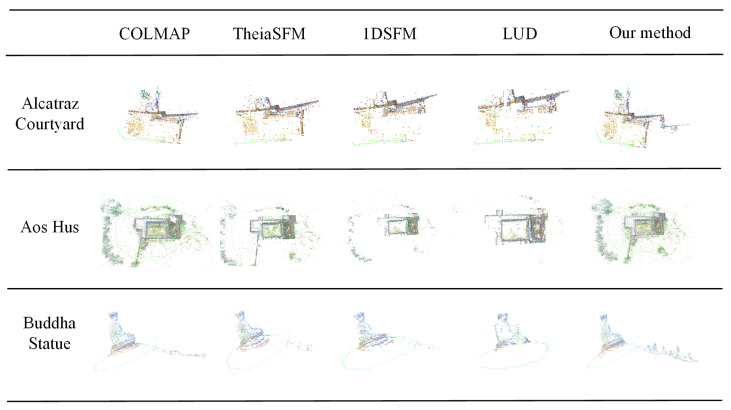
Reconstruction results on publicdatasets. From top to bottom: the Alcatraz Courtyard, Aos Hus, and Buddha Statue datasets.

The sparse point cloud reconstruction results are shown in Figure 10. From top to bottom, the datasets are Alcatraz Courtyard, Aos Hus, and Buddha Statue. Left to right in each row are the sparse point cloud results generated by COLMAP [9], TheiaSfM [70], 1dSfm [62], LUD [26], and our method. The results of point cloud visualization can also be used to verify the accuracy of the methods. As shown in Figure 10, our method can always obtain more accurate sparse point cloud results.

### 5.2. Large-Scale Datasets

**Figure 11:**
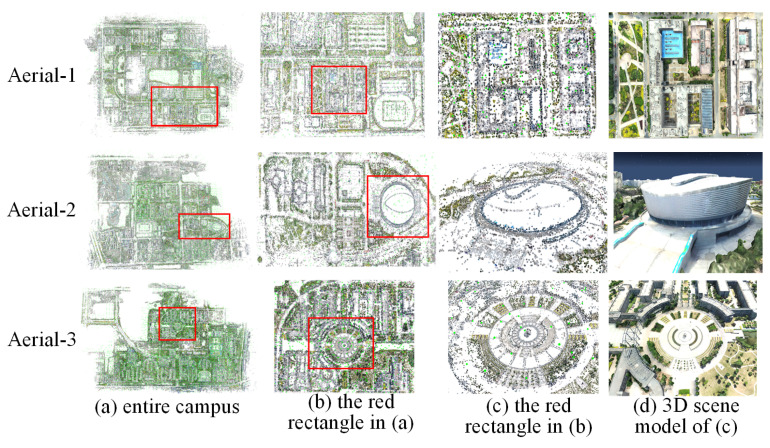
Sparse point cloud reconstruction results in large-scale scenes.

Effectiveness evaluation: We used the large-scale scenes in Section 4.2 to verify the effectiveness of our method. Due to the huge set of input images for large-scale scenes, we used all of the computing nodes in our cluster for experiments. Figure 11 shows the sparse point cloud reconstruction results for large-scale scenes. The first and second rows illustrate the scenes of datasets 1 and 2 in Section 4.2, respectively. The last row shows the campus of Tianjin Normal University. As seen in Figure 11, (a) represents the sparse point cloud reconstruction results of the entire campus, (b) represents the sparse point cloud of the red rectangle in (a), (c) is the sparse point cloud of the red rectangle in (b), and (d) is the corresponding 3D scene model of (c). As shown in Figure 11, our method can generate accurate sparse point cloud results and the corresponding 3D models for large-scale scenes.

**Performance evaluation:** Similar to the performance evaluation in Section 5.1, we compare our method with other traditional methods on large-scale datasets. Table 4 presents a comparison of the reconstruction results with COLMAP [9] and 1dSfm [62] on large-scale datasets. The three aerial datasets in Table 4 correspond to rows 1–3 in Figure 11. Although our system uses global SFM, our method and COLMAP [9] can complete almost the same number of camera registrations, and the reconstruction speed is nearly 20 times faster than that for COLMAP [9]. In addition, our system can obtain more 3D points than the other two methods.

### 5.3. Evaluations

Parameters: Although our method involves many parameters, most of these parameters are insensitive to different large-scale scenes. We used the same parameter values for all scenes throughout our experiments. All parameter values were specified in each step.

Robustness: We generated a sparse point cloud of a large-scale scene to evaluate the robustness of our method. Calculating all camera rotation and translation matrices at one time consumes a considerable amount of time and surpasses the memory limit of the computing nodes. We proposed a divide-and-conquer framework to achieve global camera pose registration for large-scale scenes. Figure 8 shows the final result of performing global camera pose registration. As shown in Figure 5b, the sparse point cloud can be generated by our method. To further validate the robustness of our approach, we generated 3D models from a sparse point cloud. As shown in Figure 11d, our approach can generate real 3D scene models.

Limitations: Although our method uses the divide-and-conquer strategy to solve the registration of camera poses in large-scale scenes, it does not improve the subsequent bundle adjustment, which still consumes a lot of computing resources. Another limitation is that the poor-quality extended cameras are discarded after the local camera pose estimation due to incomplete capture of large-scale scenes. In addition, the proposed workflow does not work when there is no available camera spatial location information.

## 6. Conclusions

We proposed a distributed 3D sparse point cloud generation method for reconstruction of large-scale scenes. By calculating the GNSS spatial neighborhood of aerial images, the square time complexity of traditional image matching was reduced to approximately linear. The divide-and-conquer framework was used to solve the distributed SFM problem. We divided the camera graph into several subgraphs and ensured that the subgraphs met the enforcement and consistency constraints. Local camera poses were merged and optimized to obtain the global camera poses. Finally, we used traditional triangulation and bundle adjustment to obtain a sparse point cloud. Compared with the traditional state-of-the-art sparse point cloud generation methods, our approach could effectively generate sparse point cloud results and the corresponding 3D models. Our method could complete the 3D sparse point cloud reconstruction of a real scene with an area of 10 km${}^{2}$ in less than 20 h. In the future, we would like to further enhance our method using global SFM and incremental SFM and to improve the subsequent bundle adjustment, which can quickly generate sparse point clouds of large-scale scenes.

## Figures and Tables

**Table 1:** Cluster configuration.

CPU	Core Number	Memory Size	Machine Number
Intel(R) Xeon(R) CPU E5-2680 v4 at 2.4 GHz	28	96	1
Intel(R) Core(TM) i7-6700 CPU at 3.40 GHz	8	32	2
Intel(R) Core(TM) i7-8700 CPU at 3.20 GHz	12	64	5

**Table 2:** Time-consuming analysis for image matching (min).

Aerial Dataset	Image Number	Traditional Method (SIFT)	Our Method on Single Computer	Our Method on a Cluster
Dataset1	1510	41.2	36.6	18.9
Dataset2	8876	2927.3	1146.1	197.6
Dataset3	25,726	24,517.5	3094.1	438.8

**Table 3:** Accuracy and efficiency evaluation with datasets having different scales. $N_{c}$, $N_{p}$, and $T_{\sum }$ represent the number of recovered cameras, number of 3D points, and reconstruction time (s), respectively.

Dataset	Images	COLMAP [9]			1DSFM [62]			LUD [26]			Ours		
		$N_{c}$	$N_{p}$	$T_{\sum }$	$N_{c}$	$N_{p}$	$T_{\sum }$	$N_{c}$	$N_{p}$	$T_{\sum }$	$N_{c}$	$N_{p}$	$T_{\sum }$
Courtyard	133	133	12,763	170.39	133	8597	113.10	132	9603	108.64	133	11,346	106.1
Aos Hus	811	801	293,541	1963.14	763	279,154	1097.46	782	230,654	984.66	800	301,449	789.18
Buddha	321	321	109,833	527.32	316	84,546	419.63	316	86,459	423.11	321	109,431	409.16
Cathedral	1227	1223	510,369	3497.24	1163	482,164	3095.60	1150	451,319	3018.76	1154	516,797	3042.84
Palace	241	234	70,468	597.91	231	66,157	519.49	231	63,149	544.50	234	70,997	497.1
Forum	1084	1083	41,064	3054.46	1076	384,988	2874.13	1075	375,556	2943.13	1083	449,731	2849.83

**Table 4:** Comparison of reconstruction results. $N_{c}$ and $N_{p}$ represent the number of recovered cameras and number of 3D points, respectively. $T_{\sum }$ denotes the total time (s), and $T_{d}$ denotes the total time (s) that is evaluated in a distributed system.

Dataset	Images	COLMAP [9]			1DSFM [62]			Ours			
		$N_{c}$	$N_{p}$	$T_{\sum }$	$N_{c}$	$N_{p}$	$T_{\sum }$	$N_{c}$	$N_{p}$	$T_{\sum }$	$T_{d}$
Aerial-1	7063	6616	3,269,946	97,461.16	6134	2,849,849	19,613.18	6614	3,319,946	9184.33	2493.18
Aerial-2	9238	8343	3,849,478	164,941.07	8097	3,349,818	31,564.62	8448	3,948,163	27,199.16	7413.94
Aerial-3	11,194	9431	4,984,134	211,496.91	8989	4,316,413	51347.41	9524	5,046,491	43,186.19	13,486.60

## Data Availability

The data presented in this study are available on request from the corresponding author.
